# SALUS—a non-inferiority trial to compare self-tonometry in glaucoma patients with regular inpatient intraocular pressure controls: study design and set-up

**DOI:** 10.1007/s00417-022-05759-7

**Published:** 2022-07-22

**Authors:** Kristina Oldiges, Maren Steinmann, Juliane Andrea Duevel, Sebastian Gruhn, Raphael Diener, Martin Dominik Leclaire, Sami Al-Nawaiseh, Nicole Eter, on behalf of the SALUS study group, W. Greiner, B. Behm, D. Kisielinski, K. Schwarze, F. Meyer, S. Warkentin, R. Hammerschmidt, M. Luzius, T. Berlage, M. Becker, A. Charles, R. Heitkaemper, B. Weingarten, T. Boeker, M. Hermel, S. Kaskel-Paul, M. Kohlhaas, M. Alnawaiseh, V. C. Brücher, P. Czapski, L. Holtrup, R.-L. Merté, J. J. Storp, M. Treder, J. A. Zimmermann

**Affiliations:** 1 Department of Ophthalmology, University of Muenster Medical Center, Albert-Schweitzer-Campus 1, Building D15, 48149 Muenster, Germany; 2 Department for Health Economics and Health Care Management, School of Public Health, Bielefeld University, Universitaetsstrasse 25, 33615 Bielefeld, Germany

**Keywords:** Glaucoma, Self-tonometry, Non-inferiority trial, Intraocular pressure, Data transfer, Reading center

## Abstract

**Purpose:**

The SALUS study aims to improve the healthcare situation for glaucoma patients in Germany. In order to detect diurnal intraocular pressure (IOP) fluctuations, inpatient monitoring of IOP in an eye hospital for a minimum of 24 h is the current standard. SALUS assesses the benefits of a new form of outpatient care, where IOP can be measured by the patients themselves at home using a self-tonometer. This approach should promote the patient’s health competence and empowerment within the healthcare system while reducing treatment costs.

**Methods:**

The SALUS study is a randomized controlled, open non-inferiority trial, alongside an economic analysis, determining whether outpatient monitoring of IOP with self-tonometry is at least as effective as current standard care and would reduce treatment costs. Participants (*n* = 1980) will be recruited by local ophthalmologists in the area of Westphalia-Lippe, Germany, and randomized to receive 7-day outpatient or 24-h inpatient monitoring. Participants in both study arms will also receive 24-h blood pressure monitoring. Furthermore, patient data from both study groups will be collected in an electronic case file (ECF), accessible to practitioners, hospitals, and the study participants. The primary endpoint is the percentage of patients with IOP peaks, defined as levels 30% above the patient-specific target pressure. Data will also be collected during initial and final examinations, and at 3, 6, and 9 months after the initial examination.

**Results:**

The study implementation and trial management are represented below.

**Conclusion:**

SALUS is a pioneering prospective clinical trial focused on the care of glaucoma patients in Germany. If SALUS is successful, it could improve the healthcare situation and health literacy of the patients through the introduction of various telemedical components. Furthermore, the approach would almost certainly reduce the treatment costs of glaucoma care.

**Trial registration:**

*ClinicalTrials.gov ID:* NCT04698876, *registration date:* 11/25/2020.

*DRKS-ID:* DRKS00023676, *registration date:* 11/26/2020.


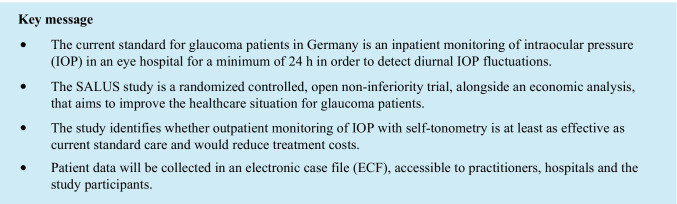


## Introduction

Glaucoma is one of the most common chronic eye diseases with almost one million affected individuals in Germany and rising tendency, as the risk of glaucoma increases with age [1]. The disease results in an irreversible loss of optic nerve fibers with associated progressive visual field damage, leading to complete blindness if left untreated or poorly treated [1]. Currently, glaucoma is still one of the leading causes of blindness in Western industrialized countries [1]. It is hypothesized that an elevated intraocular pressure (IOP) and a lack of blood supply to the optic nerve head both contribute to the progressive loss of retinal ganglion cells. Though sophisticated imaging techniques, like optical coherence tomography (OCT) of the optic nerve head with assessment of the retinal nerve fiber layer (RNFL) thickness and of Bruch’s membrane opening minimum rim width (BMO-MRW) and, more recently, OCT angiography (OCTA), are available to diagnose and assess glaucoma progression, the IOP is still the only direct control parameter of therapy response.

Studies show that lowering the IOP reduces the rate of glaucoma progression and decreases the frequency of a progression from ocular hypertension to glaucoma [1–3] Typically, IOP controls in glaucoma patients are performed three to four times per year. However, the IOP as a control parameter can be subject to large fluctuations with pressure peaks during the day, and such pressure fluctuations are especially common in glaucomatous eyes [4, 5]. Prior to the start or adjustment of therapy, measurement of IOP in hospitals at 4-h intervals over several days (minimum 24 h) can be helpful, especially in the case of disease progression despite normal IOP measured on an outpatient basis or in the case of unclear optic nerve disorders. Goldmann applanation tonometry (GAT) and rebound tonometry are commonly used. The measurements are usually performed by doctors or other medically qualified personnel. Several studies have confirmed the importance of 24-h IOP monitoring, demonstrating that more than half of all glaucoma patients have their peak IOP outside conventional working hours [6, 7]. A study comparing 24-h inpatient IOP measurements with those taken during normal working hours showed that implementing 24-h IOP monitoring led to a change in clinical management in 79.3% of patients [8].

The new, commercially available IcareHOME® self-tonometer enables patients to take their own IOP measurements. This could reduce the need for inpatient stays. Evaluation of data from these devices by the attending ophthalmologist nevertheless ensures professional support of the patients and enables therapy planning to be based on the data collected. However, self-tonometry has not yet been incorporated into standard care in Germany.

Termühlen et al. demonstrated comparable IOP values in GAT and self-tonometry. In addition, the taking of measurements by the physician and by the patient were found to be comparable [9]. Even though some patients were more than 75 years old, their rating of the self-tonometer in the categories usability, feeling of safety, and comfort lay between “satisfied” and “very satisfied.” The time taken for an average measurement was between 45 s (younger patients) and 88 s (older patients) for both eyes [10].

Pilot projects for telemedical home monitoring of IOP have been carried out at the University Eye Hospital Greifswald with positive effects on care [11, 12]. Dietlein et al. analyzed the data of 130 glaucoma patients and concluded that an increase in understanding of the disease, better motivation to increase compliance, and more practical involvement of patients are necessary [13, 14]. Lämmer et al. concluded in their study that the compliance generated by the close doctor-patient relationship within telematically assisted self-tonometry is a positive prognostic factor for glaucoma [11].

Based on these findings, the overall objective of the SALUS study, standing for “Self Tonometry and Transfer of Glaucoma Patients’ Data for Improving the Supply Situation,” is to determine the effectiveness, acceptability, and reliability of outpatient monitoring of IOP using an IcareHOME® self-tonometer, compared to the existing standard procedure in Germany. Since self-tonometry does not require admission to hospital, this new approach could potentially reduce costs and improve the quality of care for glaucoma patients.

More specifically, the SALUS study aims to demonstrate that self-tonometry is non-inferior to the established 24-h inpatient IOP measurements for monitoring and treating glaucoma. The primary hypothesis in this non-inferiority trial is that the percentage of patients in whom pressure peaks could be detected during the pressure profile would not differ significantly between the intervention group (IG = outpatient self-tonometry) and the control group (CG = inpatient measurement every 3 h over the course of 24 h). In this study, a pressure peak was defined as a level 30% above the patient-specific target pressure which will be set by the treating ophthalmologists. The health economic hypothesis tests whether the use of self-tonometers reduces costs from a healthcare provider perspective compared to current standard care.

## Materials and methods

### Study design

The SALUS study is a multicenter, two-arm parallel group, open non-inferiority, randomized controlled trial with an economic analysis alongside.

**Fig. 1:**
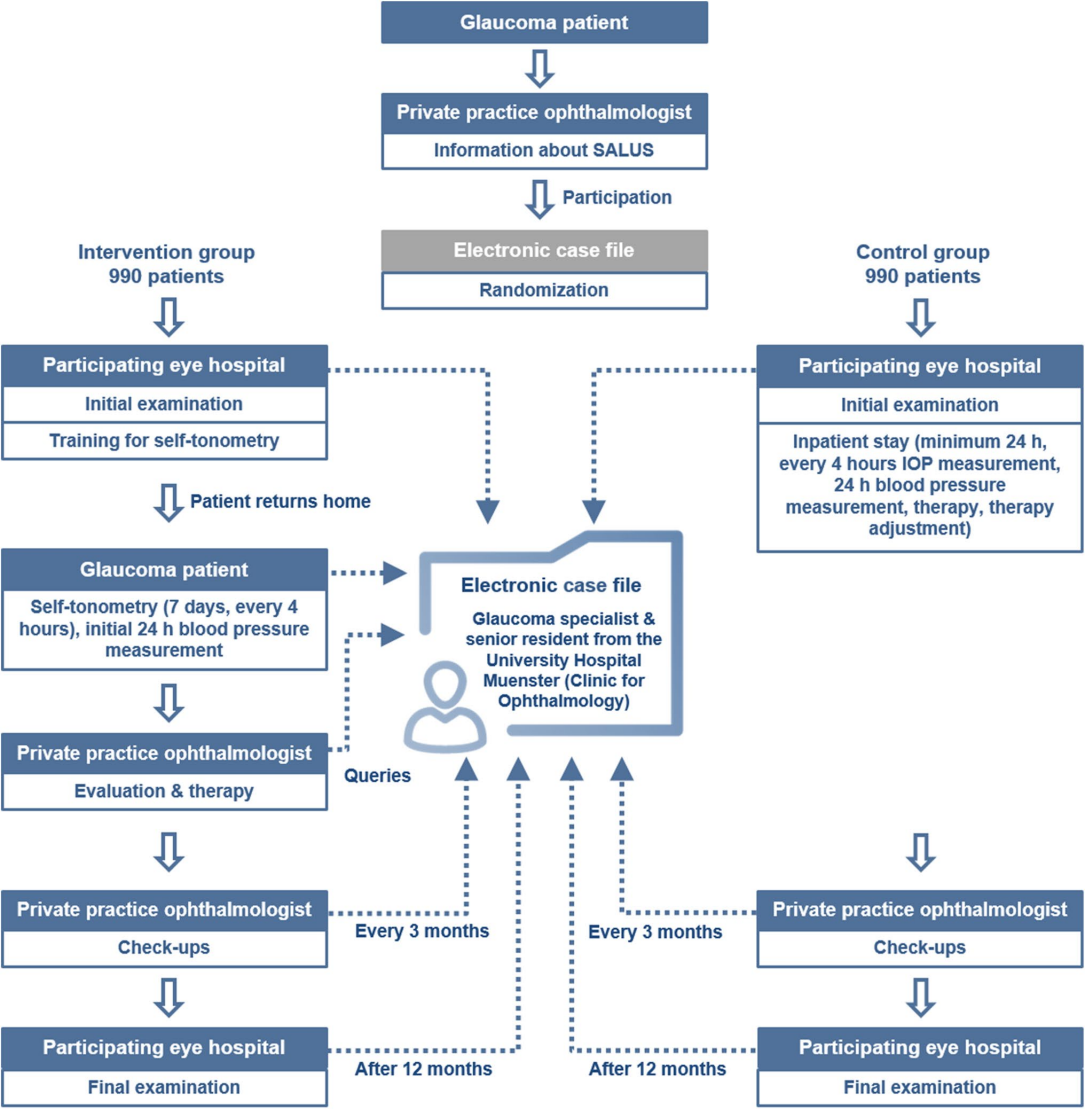
Study flow chart

This non-inferiority design was chosen to establish that outpatient monitoring of IOP with self-tonometry is not less effective than standard care with hospitalized monitoring of IOP. The trial will be conducted in hospitals and patients’ homes in the district of Westphalia-Lippe, Germany. Each participant will be followed for 12 months from the start of the trial, with regular follow-up every 3 months by local ophthalmologists (see Fig. 1).

### Intervention

The main part of the intervention is outpatient monitoring of IOP using a self-tonometer. The glaucoma patients who qualify to participate in the study will be briefed by the local ophthalmologist about the study design as well as the benefits and risks and asked to sign an informed consent form. After randomization, they will be contacted by the Department of Ophthalmology of the University of Muenster Medical Center to arrange an appointment at one of the participating eye hospitals for an initial examination.

The initial examination includes IOP measurement to investigate the pathological pressure peaks, a 30° visual field examination, an OCT of the optic nerve head, a Heidelberg retina tomography (HRT) (if available), and a questionnaire about health-related quality of life. Subsequent to the initial examination, patients will be instructed by the clinical staff in the functioning and handling of the IcareHOME® self-tonometer and 24-h blood pressure measuring device. The patients of the IG will then receive the devices and perform self-tonometry over the course of seven days. They will also undergo initial blood pressure monitoring for 24 h, starting at the end of the training. The IOP and blood pressure values measured will be evaluated by the local ophthalmologists, who can adjust the patient’s therapy on the basis of the results. Thereafter, check-ups will be carried out every 3 months by the local ophthalmologists. The final examination will be performed 12 months after the initial examination at the same participating eye hospital and will replicate the initial one but end with a final interview between the doctor and patient.

All measured values, the medical history, and the imaging performed will be transferred by the local ophthalmologists or hospitals to an electronic case file (ECF), a cloud-based system for the management of the patient’s primary data. The ECF can also be used to raise queries with a glaucoma specialist and senior resident from the University of Muenster Medical Center. IG participants will also have access to the ECF and be able to view all measurement data. The participants will gain a comprehensive insight into their disease progression for the first time and will also become more involved and informed about treatment options.

### Study population, sampling strategy, and eligibility criteria

All patients with confirmed or suspected glaucoma who require inpatient day and night IOP monitoring within the state of Westphalia-Lippe will be invited to participate in this study. Confirmed or suspected glaucoma will be classified according to the international ICD-10 classification (see Table 1). Patients eligible to participate in SALUS will be identified by their local ophthalmologists during initial consultations for glaucoma or routine check-ups. Fulfilment of inclusion or exclusion criteria (Table 1) and recruitment will be established by the ophthalmologists based on their clinical experience. Eligible patients, willing to participate, will receive an information sheet explaining details of the study and the terms intervention and randomization and be asked to provide written informed consent before randomization.

**Table 1:** Inclusion and exclusion criteria

Inclusion criteria	Exclusion criteria
I. 18 years of age or older	I. Younger than 18 years of age
II. Requiring inpatient day and night IOP monitoring	II. Patients outside of the usual catchment area of the participating hospitals
III. Statutory health insurance	III. Concomitant psychiatric or neurological diseases or other conditions limiting the ability to perform self-tonometry independently
IV. Confirmed or suspected glaucoma with pressure variations and peaks, failure to achieve intraocular target pressure or progression of glaucoma (ICD-10 H40.0 Glaucoma suspect, H40.1 Primary open-angle glaucoma, H40.2 Primary angle-closure glaucoma and H42.- Glaucoma in diseases classified elsewhere)	
V. Willingness to be admitted to one of the participating hospitals as an inpatient	IV. Strong barriers to communication that would not allow informed consent or understanding of patient information
VI. Sufficient knowledge of German	
VII. Signed and dated informed consent for study participation and data transfer	V. Unclear legal capacity to agree to participate in the study
VIII. Legal capacity to agree to participate in the study	

### Randomization

After informed consent, patients will be randomized 1:1 into the IG and CG. The randomization will be generated automatically by the ECF after the local ophthalmologist has submitted the patient’s master data (year of birth, sex, and three digits of the zip code) and eligibility criteria in a web-based form. The underlying randomization sequence will be known only to the developers of the ECF (Fraunhofer Institute for Applied Information Technology FIT) who will act independently of the personnel analyzing (Bielefeld University) and conducting (University of Muenster Medical Center) the study. This sequence will be concealed from all study staff so that it cannot be manipulated by physicians, clinical staff, or the patients themselves.

Participants will receive a study identification number after randomization and their assignment to one study group. Owing to the nature of the intervention, it will not be possible to blind either the patients, the personnel conducting the study or the healthcare providers. The independent evaluators of the study will be blinded during the study by being provided solely with a pseudonymized data set for data analysis.

### Primary endpoint

The primary endpoint is the percentage of detected IOP peak values. Fluctuations and peak values of IOP are considered as a patient-relevant outcome that can be associated with glaucoma progression and warrant adjustment of therapy [15–18].

In the IG, the primary endpoint will be measured by the patients themselves for each eye using an IcareHOME® self-tonometer at six predefined times (6 a.m., 8 a.m., 12 a.m., 4 p.m., 8 p.m. and 12 p.m.) for 7 days (see Table 2). The measurements at 6 a.m. and 12 p.m. will count as night measurements. The night measurements are performed in sitting position, preferably without standing up before. The exact time is recorded electronically. One measurement sequence is based on six individual readings. The IcareHOME® devices calculates the final IOP measurement by discarding the highest and lowest readings and only displaying the mean of the remaining four readings [19]. In the CG, clinical staff will measure the IOP of the patients over at least 24 h at the same times of day and night using rebound tonometry or GAT. To compare these measurements with the IcareHOME®, six readings will be obtained for each eye and the mean of these values will be used for analysis, after discarding the highest and lowest readings.

**Table 2:** Study endpoints

Study endpoints	Instruments	Time of assessment						
		*T*_−1_	*T*_0_	*T*_1_	*T*_2_	*T*_3_	*T*_4_	*T*_5_
Patient-related endpoints								
Health-related quality of life	EQ-5D questionnaire		X					X
Clinical endpoints								
IOP fluctuation and peak values	Outpatient/inpatient IOP monitoring	X	X	X*	X	X	X	X
VFI	Standard automated perimetry		X				X	X
MD	Standard automated perimetry		X				X	X
PSD	Standard automated perimetry		X				X	X
Retinal nerve fiber layer thickness	OCT/HRT		X					X
Rim volume	OCT/HRT		X					X
Rim area	OCT/HRT		X					X
Blood pressure (RR)	24 h blood pressure measurement			X*				
Economic endpoints								
Resource utilization (outpatient and inpatient)	Service providers/statutory health insurers	12-month course						
Costs	Service providers/statutory health insurers	12-month course						
Days of incapacity to work (unspecific and diagnosis-specific)	Surveys/statutory health insurers	12-month course						
Sick pay days	Surveys/statutory health insurers	12-month course						
Formative assessment								
Implementation-promoting and inhibiting factors	Interviews, questionnaire	During or at end of project						
Usability	Interviews, questionnaire	During or at end of project						

^*^Measurement of IOP and blood pressure will be performed either during inpatient stay in participating hospitals (CG) or at home (IG. The blood pressure monitor will be put on in hospital and should be worn at home, while self-tonometry is performed by patients themselves over a time frame of 7 days)

*Time stamps:*

*T*_*−1*_*:* Enrolment of patients by local ophthalmologists

*T*_*0*_*:* Initial examination in a participating hospital

*T*_*1*_*:* For IG: consultation with local ophthalmologist for evaluation of data from self-tonometry and 24-h blood pressure measurement; for CG: hospital admission for IOP measurements and 24-h blood pressure measurements

*T*_*2*_*:* First check-up examination at a local ophthalmology practice after 3 months

*T*_*3*_*:* Second check-up examination at a local ophthalmology practice after 6 months

*T*_*4*_*:* Third follow-up examination at a local ophthalmology practice after 9 months

*T*_*5*_*:* Final examination in a participating hospital

### Secondary endpoints

#### Clinical endpoints

Visual field indices such as visual field index (VFI), mean deviation (MD), and pattern standard deviation (PSD) will be determined using standard automated perimetry. These values will be measured on initial and final examination in hospitals and after 9 months during the follow-up visit at the local ophthalmology practice. These measurements will be used to determine the extent of visual field loss due to suspected or confirmed glaucoma. Furthermore, the values will be used to calculate visual field damage and classify stages of glaucoma [20, 21].

RNFL thickness, rim volume, and rim area will be needed to map short- and medium-term results of glaucoma diagnosis and progression within the project time-frame [22]. Those surrogates will be determined with OCT (RNFL thickness) and HRT (rim volume and area), if available, during initial and final examination in participating hospitals.

IOP will be measured on all examinations in participating hospitals and at the local ophthalmology practice. Since low ocular perfusion pressure and low blood pressure are associated with an increased risk of glaucoma, vascular factors can be of importance for further glaucoma treatment planning [23]. Measurement of blood pressure will be performed parallel to IOP monitoring during inpatient stay or at home for 24 h, every 15 min during day between 6 a.m. and 10 p.m. and every 30 min at night between 20 p.m. and 6 a.m.

Health-related quality of life will be evaluated using the EQ-5D-5L questionnaire, a generic multidimensional instrument implemented by the EuroQol Group [24, 25]. The EQ-5D is frequently used to analyze quality of life in ophthalmic diseases such as glaucoma, and glaucoma and disease progression with visual damage are known to influence health-related quality of life [26–29].

#### Health economic endpoints

Costs of healthcare services as well as resource utilization for both groups will be estimated on the basis of secondary data from the statutory health insurers participating in the SALUS study. More precisely, this will include direct costs such as those of general and specialist care from inpatient and outpatient visits, medication, therapeutic products, medical aids, and other health services related to suspected or diagnosed glaucoma. Costs associated with absenteeism will also be analyzed. Those data will be provided by the statutory health insurers. Cost effects and differences between IG and CG can then be analyzed, as well as the costs related to glaucoma progression [30, 31].

#### Formative assessment

In addition, patient-related usability and user acceptance of the IcareHOME® tonometer as well as usability and user acceptance of the ECF for healthcare providers and participants will be assessed through interviews and a questionnaire, based on the Unified Theory of Acceptance and Use of Technology (UTAUT) model [32], which will examine expectancy, effort expectancy, social influence, and facilitating conditions.

### Sample size

The sample size was determined using the license-free software “R” (R v. 3.6.3, Rstudio v. 1.2.5042). The estimation of sample size and power analysis is based on the primary endpoint “exceeding patient-specific target pressure”. As null hypothesis, it is assumed that the proportion of pressure peaks in IG is smaller than the proportion in CG (i.e., non-inferiority).

**Fig. 2:**
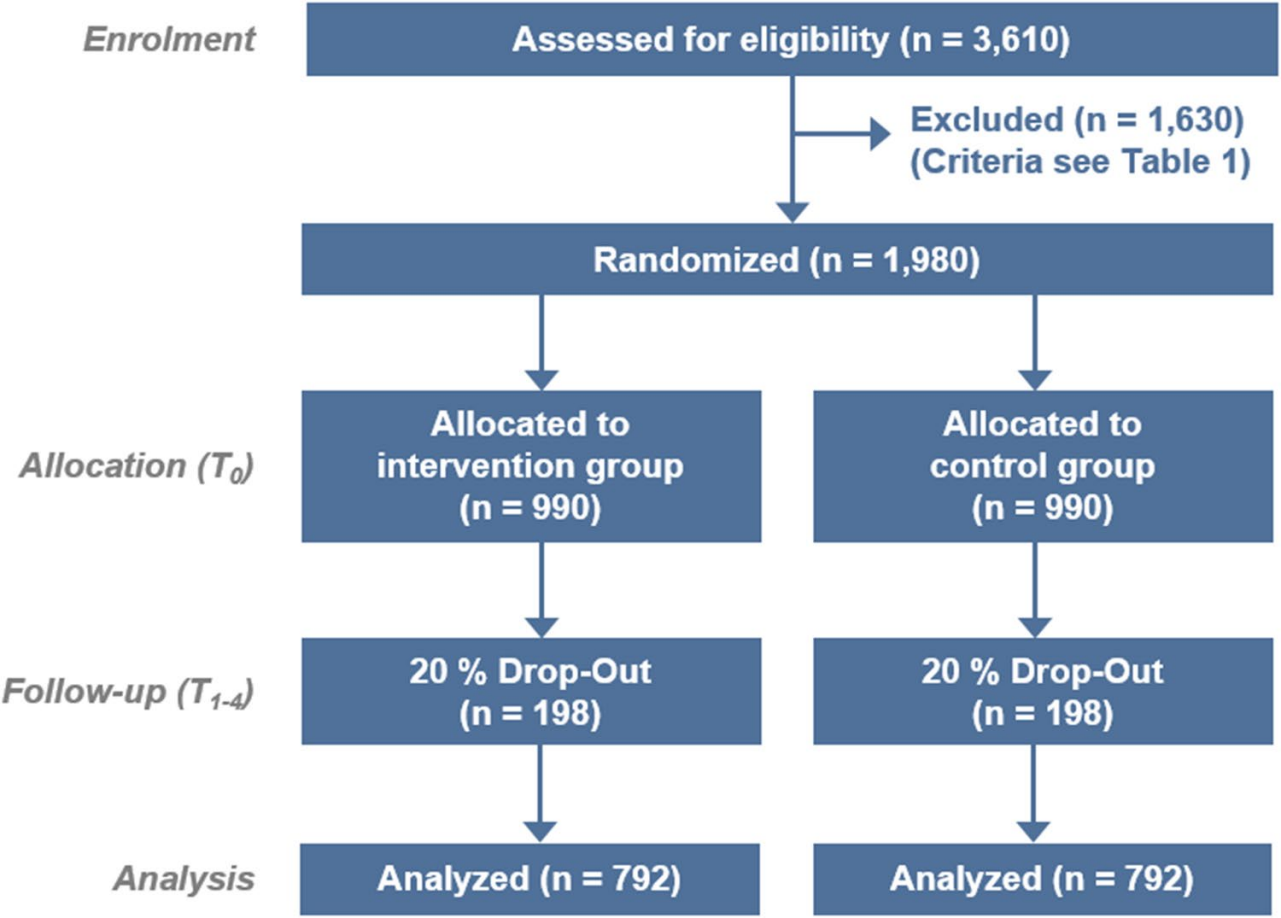
CONSORT statement

Based on clinical experience, a margin of 20% is assumed for detection of IOP fluctuations and peak values in both groups. A non-inferiority margin of *δ* = 0.05 is acceptable, meaning the proportion of pressure peaks in self-tonometry should fall below the proportion in 24 h IOP by more than 5% to be taken as a statistically significant difference [34, 35]. With regard to the probability of type I and II errors, significance levels will be set at *α* = 0.05 and *β* = 0.2, respectively, i.e., a power of (1 − *β*) = 0.8. This results in a total of 1584 patients (792 per group). After adjusting for a drop-out rate of 20%, a final sample size of 1980 patients (990 per group) will be required for this trial (see Fig. 2).

## Results

### Selection and recruitment of local ophthalmologists and patients

Since local ophthalmologists will assess the need for the intervention, they will decide which patients to include in the study. The ophthalmologist will inform all patients who would receive inpatient monitoring of IOP as standard care about the SALUS study. There will be no selection based on severity of illness or criteria other than the inclusion and exclusion criteria of the study (Table 1).

In general, every ophthalmologist in Westphalia-Lippe will have the opportunity to participate in the project. The recruitment of ophthalmologists appears to be particularly effective with a good cost–benefit ratio. Those with a high number of glaucoma patients are preferentially recruited, not least because such practices are likely to enroll the most patients in the study. The work involved should be communicated to the ophthalmologists in an easily understandable and concise manner, while any further effort on their part should be kept to a minimum. The ophthalmologist should be able to access information at self-selected times. The trial will be brought to the attention of the ophthalmologists mainly via e-mails, letters, information events, website, and articles. Participating ophthalmologists will get a detailed study folder with all relevant documents and a guideline for briefing the glaucoma patients to make the procedures standardized. They can also attend optional training sessions.

Patients will be informed by project flyers or addressed directly by the physicians. An information hotline will be available on weekdays for all interested parties. High drop-out numbers are not expected for patients assigned to the CG, as they will receive services that are current standard care. There is just one additional appointment at the end of the study period, which is the final examination in the eye hospital. It is assumed that the patients will take advantage of this, on account of the intensive care and additional review of their disease and medication, which could help to avoid further complications and the risk of blindness.

### Data management and monitoring

The ECF has been developed for collecting and managing the patient’s primary data that can be accessed by the local ophthalmologists, hospitals, and the study participants. The eye hospitals will be responsible for the care of their regionally assigned patients. As stated above, the patients will be automatically randomized and patient-specific informed consents will be created by the system.

At predefined points in time, the primary data collected in the ECF and secondary data provided by the participating health insurance companies, both required for evaluation of the study, will be transmitted end-to-end in encrypted form to the trust center at Bielefeld University, which will act independently from the evaluating center. The data will then be pseudonymized, merged, and forwarded to the evaluating center.

For quality assurance, monitoring and grading of images and data will be necessary and will require evaluation by several physicians at a reading center. Typically, one physician will act as junior reader and the other as senior reader to uphold the four-eye principle. In case of doubt, reader A and reader B can each serve as junior reader. In the SALUS project, a glaucoma specialist and a senior resident will be responsible for the grading of diagnostics. During grading, the OCT and HRT images (if available) from the initial and final examination after 12 months will be evaluated with regard to the required outcome parameters (RNFL, BMO-MRW) for each patient group. In addition, the completeness and plausibility of further examination results from the hospital and from the participating local ophthalmologist (IOP, blood pressure, perimetry) will be checked.

### Statistical analyses

All statistical analyses will be performed using the current version of R/Rstudio and results will be presented following the CONSORT recommendations [36, 37]. Baseline characteristics (including sociodemographic and clinical data) will be analyzed using appropriate descriptive methods. These will include absolute and relative frequency distributions and measures of dispersion, such as median, mean, quantile, variance or standard deviation, used as appropriate, and visualized in a suitable form (e.g., boxplot or histogram). More precisely, baseline characteristics will be summarized for the IG and CG. Standard deviation (when assuming a Gaussian distribution), median, and interquartile ranges will be applied for continuous data (e.g., VFI or blood pressure) and frequencies or percentages will be used for binary data. After analyzing the entire cohort, subgroup analyses will be conducted to determine potential effect modifications (e.g., time effects, age or gender) on the primary endpoint.

The primary endpoint, percentage of detected peak values in IOP, will be analyzed using suitable inferential statistic methods (e.g., Mann–Whitney-*U* test for independent samples and assuming a normal distribution or odds ratios for binary outcomes). An appropriate regression model will be used to evaluate which regressor has an influence on the primary endpoint and residual analysis will be applied to test the results of the model for significance. Secondary endpoints will be evaluated using a similar analysis strategy while using appropriate models for the outcomes.

Data will be analyzed on the intention to treat principle (ITT). Thus, all participants who have given their informed consent will be included in the evaluation. More precisely, all patients of the IG and CG will need to be informed about the use of their data, and their willingness to participate must be ascertained and documented. Allocation to IG and CG as well as randomization will be conducted independently after recruitment of the study participants to ensure structural equality of the data between the two groups. To maintain this structural equality for data analysis, all participants will be evaluated according to the ITT principle as members of the treatment group to which they were randomized, whether or not the study participants used the allocated outpatient or inpatient method of measurement. Further, the potential amount and pattern of missing data will be defined, and if required, appropriate methods such as sensitivity analysis will be applied. This will allow accurate analysis of the assumptions and the effectiveness of self-tonometry in this study [38–40]. All models implemented will provide information on potential treatment effects and will be presented as a 95% confidence interval.

Following the recommendation of the German Institute for Quality and Efficiency in Health Care (IQWiG), the cost-minimization analysis will be carried out from the perspective of statutory health insurance and will, if applicable, be supplemented by analysis from a patient/societal perspective [41]. The economic analysis will conform to the principles of good secondary data analysis, the recommendations of the memorandum “Methods for Health Services Research,” and the standards of the German Evaluation Society [42–44].

## Discussion

The SALUS study is the first randomized, controlled prospective clinical trial to investigate the effectiveness of self-tonometry. The healthcare situation of glaucoma patients could be improved by the measuring of IOP over several days, especially in rural, structurally disadvantaged areas and those with fewer specialist health services.

In this study, patient-input is integrated into the diagnosis and treatment plan. It gives patients comprehensive insights into the course of their disease and promotes compliance. If self-tonometry is established in regular patient care as a result of SALUS, this could reduce the need for inpatient hospitalization. On the one hand, this would free up capacity in hospitals, and on the other hand, it would provide more comfort for patients, i.e., bring benefits without requiring extra effort or absence from work. The fact that patients could then remain in their accustomed domestic environment and daily routine is expected to improve their quality of life. They may save travel costs and would not be unnecessarily exposed to the risk of infection, especially in times of a pandemic. The taking of IOP measurements at a series of times predefined by the study will enable therapy to be optimally adjusted, and complications arising from delayed adjustments to therapy or more complex operations could possibly be avoided. The long-term benefit could be less blindness due to glaucoma. The telemedical focus could significantly improve healthcare by making medical services available independent of time and space. Telemedical networking via the cross-institutional ECF could optimize collaboration between the areas of healthcare provision and service providers. The ophthalmologist could rely on the support of continuously improving, self-learning algorithms in the evaluation of findings and therapy decisions, thus optimizing treatment strategies. Through digital networking, repeat medical examinations could be avoided when treatment of a patient takes place in another treatment center. The local ophthalmologists will be professionally supervised by a glaucoma expert and a senior resident and have the opportunity to base their treatment on an improved database.

Healthcare costs could be reduced by saving hospital resources. Once incorporated into standard care, economies of scale could lower prices further. Moreover, the fixed and implementation costs could be spread over a greater number of patients.

In summary, the SALUS study could be of great benefit to all parties involved, i.e., hospitals, patients, and also local ophthalmologists. In a further step, long-term comparative controls are necessary to assess the progression of glaucoma and further evaluate the reliability of self-tonometry. The focus on telemedicine is forward-looking and in line with current healthcare trends.

## Data Availability

Not applicable.

## Appendix

**SALUS Study Group (in alphabetical order)**

*Principal investigator*: N. Eter (University of Muenster Medical Center).

*Project manager*: K. Oldiges (University of Muenster Medical Center).

*Evaluators*: J. A. Duevel, W. Greiner (principal evaluator), S. Gruhn, M. Steinmann (all Bielefeld University).

*Other members of the consortium:*

B. Behm (DAK-Gesundheit), D. Kisielinski (BARMER), K. Schwarze *(*AOK NordWest), F. Meyer, and S. Warkentin (IKK classic) (all statutory health insurers).

R. Hammerschmidt and M. Luzius (Association of Statutory Health Insurance Physicians Westphalia-Lippe, KVWL), T. Berlage (Fraunhofer Institute for Applied Information Technology FIT).

*Cooperation partners*:

M. Becker (KKH Kaufmännische Krankenkasse), A. Charles (VIACTIV), R. Heitkaemper (KNAPPSCHAFT), B. Weingarten (Techniker Krankenkasse) (all statutory health insurers).

T. Boeker (Klinikum Dortmund), M. Hermel (Katholisches Krankenhaus Hagen), S. Kaskel-Paul (Klinikum Lüdenscheid), M. Kohlhaas (St.-Johannes-Hospital Dortmund) (all eye hospitals).

*Other contributors from University of Muenster Medical Center*: M. Alnawaiseh, S. Al-Nawaiseh, V. C. Brücher, P. Czapski, R. Diener, L. Holtrup, M. D. Leclaire, R.-L. Merté, J. J. Storp, M. Treder, J. A. Zimmermann.
